# Neutrophils in oncolytic virus immunotherapy

**DOI:** 10.3389/fimmu.2024.1490414

**Published:** 2024-12-04

**Authors:** Danya Zhou, Chenglin Zhang, Jingyi Sun, Ming Yuan

**Affiliations:** ^1^ Department of Dermatology, The First Affiliated Hospital of Anhui Medical University, Hefei, Anhui, China; ^2^ Key Laboratory of Dermatology (Anhui Medical University) Ministry of Education, Hefei, Anhui, China; ^3^ National Centre for International Research in Cell and Gene Therapy, School of Basic Medical Sciences, Academy of Medical Sciences, Zhengzhou University, Zhengzhou, China; ^4^ Huayao Kangming Biopharmaceutical Co., Ltd, Shenzhen, China

**Keywords:** neutrophils, oncolytic viruses, oncolytic virus immunotherapy, cancer, antiviral immune response

## Abstract

Oncolytic viruses have emerged as a highly promising modality for cancer treatment due to their ability to replicate specifically within tumors, carry therapeutic genes, and modulate the immunosuppressive tumor microenvironment through various mechanisms. Additionally, they show potential synergy with immune checkpoint inhibitors. A study report indicates that from 2000 to 2020, 49.5% of oncolytic viruses were administered intratumorally and 35% intravenously during clinical trials. However, both administration methods face significant challenges, particularly with intravenous delivery, which encounters issues such as non-specific tissue uptake, neutralizing antibody responses, and antiviral effects mediated by various immune cells. Despite extensive research into the antiviral roles of CD8+ T cells and NK cells in oncolytic virus therapy, neutrophils—constituting approximately 50% to 70% of human peripheral blood leukocytes—have received relatively little attention. Neutrophils are the most abundant leukocyte subset in peripheral circulation, known for their phagocytic activity. Beyond their traditional roles in bacterial and fungal infections, emerging literature suggests that neutrophils also play a critical role in the body’s antiviral responses. Given the gaps in understanding the role of neutrophils in oncolytic virus therapy, this article reviews current literature on this topic. It aims to provide a theoretical foundation for developing oncolytic virus-based cancer therapies and enhancing their anti-tumor efficacy in future clinical treatments.

## 1 Introduction

Oncolytic viruses (OVs) have shown significant potential in cancer therapy due to their ability to selectively replicate within and lyse tumor cells, exploiting aberrant signaling pathways and the impaired antiviral defense mechanisms in tumor cells. This selective replication activates the tumor immune microenvironment (TME) while sparing healthy cells, thereby enhancing their therapeutic potential. Various OVs have been tested in clinical trials, including Adenovirus (AdV), Vesicular stomatitis virus (VSV), Measles virus (MV), Reovirus (RV), Newcastle disease virus (NDV), Herpes simplex virus (HSV), and Vaccinia virus (VACV) (1–9).

Between 2000 and 2020, 3,233 patients received oncolytic virotherapy (OVT). This therapy not only utilizes naturally oncolytic viral vectors but also incorporates genetic modifications to reduce viral pathogenicity and enhance therapeutic efficacy. Therapeutic genes are introduced into non-essential regions of the viral genome to deliver targeted cancer therapies, promote anti-cancer activity, induce immune responses, inhibit tumor angiogenesis, and enhance radiosensitization (10). Delivery methods for OVs have evolved, with initial research focusing on direct intratumoral injection (i.t.), which has limitations for deep or metastatic tumors. As a result, intravenous (i.v.) injection has become a more viable option for targeting multiple metastatic lesions. However, challenges such as immune cell interactions in the bloodstream can affect the biological distribution of OVs and limit their efficacy.

Neutrophils, which constitute 50%-70% of peripheral blood leukocytes, are traditionally known for their role in defending against bacterial and fungal infections (11). Recent evidence indicates that neutrophils also play a significant role in antiviral responses. They can directly phagocytize viruses, release antiviral peptides like alpha-defensin Human Neutrophil Peptide-1 (HNP-1), and produce antimicrobial peptides such as Cathelicidin LL-37, which neutralize viral particles (12, 13). Emerging research suggests that neutrophils are also crucial in the context of oncolytic virus therapy. For instance, Patients with low neutrophil-to-lymphocyte ratio before treatment had significantly longer OS (P < 0.001) (14). In animal models, neutrophil depletion has impaired the antitumor effects of oncolytic measles virus (15), and significant neutrophil infiltration has been observed in tumor tissues during treatment with recombinant VACV in both mouse models and clinical trials (16, 17).

This paper reviews the role of neutrophils in various oncolytic virus therapies, providing a theoretical foundation to enhance the clinical application of these therapies and improve their antitumor efficacy.

## 2 Oncolytic viruses

### 2.1 Introduction to oncolytic viruses

In the early 1904s, it was serendipitously discovered that the influenza virus could be used to treat leukemia, sparking significant interest in the concept of oncolytic viruses (18). However, due to their nature as foreign pathogens, these viruses posed challenges in controlling toxicity and eliciting strong immune responses, which hindered their development and application. The advent of genetic engineering in the 1990s marked a transformative period for the oncolytic virus field. Genetic modifications enabled the development of oncolytic viruses with reduced toxicity and the ability to carry therapeutic genes targeting tumors, leading to a rapid advancement in the field (19). In 2004, the FDA approved the first oncolytic virus, and since then, numerous oncolytic viruses have entered clinical trials (20). Oncolytic viruses offer several mechanisms to specifically target and replicate within tumor cells, distinguishing them from other cancer treatments (21–23):

#### 2.1.1 Tumor cell surface antigen overexpression

Many types of oncolytic viruses need receptor-mediated entry into cells, and tumor antigen overexpression on the surface of cancer cells enhances the tumor targeting of oncolytic viruses. Compared to normal cells, cancer cells have high expression of receptors on their surface, such as CD46, which facilitates the targeting of oncolytic viruses such as measles virus and adenovirus to cancer cells (24, 25).

#### 2.1.2 Defective Signaling Pathways

Normal cells often have signaling pathways that inhibit viral growth that may be defective in tumor cells, allowing the virus to replicate more efficiently.

The IFN signaling pathway plays an important role in controlling normal cell growth and apoptosis, however it is deficient in tumors, which facilitates viral replication. For example, VSV has a diminished role in interferon-responsive cells and a high oncolytic role in tumor cell (26).

#### 2.1.3 Dysregulation of tumor metabolism

Tumor cells are metabolically reprogrammed to obtain more energy to meet their rapid proliferation and invasion, such as enhanced nucleic acid metabolism, protein metabolism, and glucose metabolism, which provide benefits for viral replication (23).

#### 2.1.4 Defective Apoptosis Pathways

**Figure 1:**
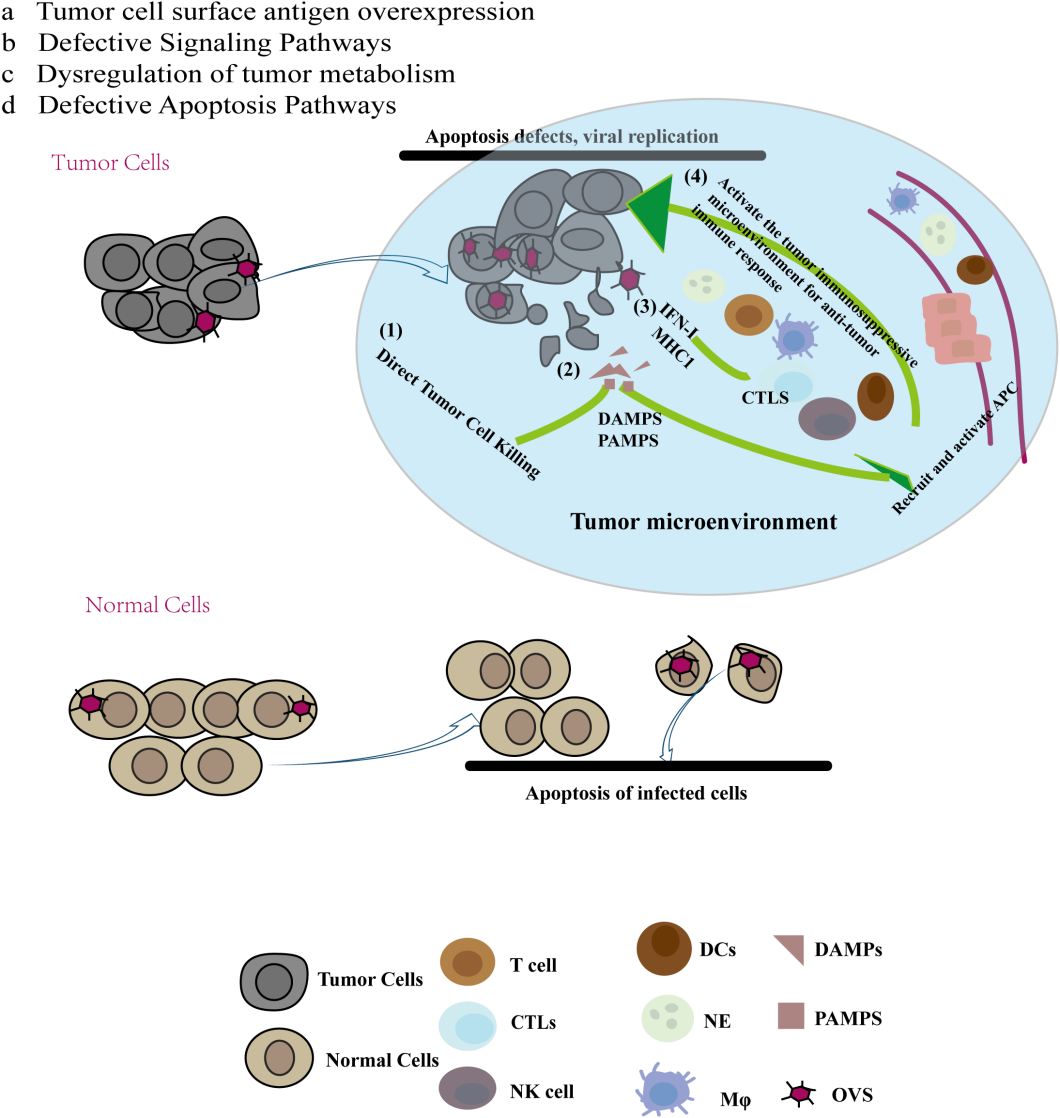
Multiple mechanisms of anti-tumor immunity of oncolytic virus (OV): Compared with normal cells, cancer cells have the specificity of abcd, and due to this specificity, oncolytic virus preferentially chooses to replicate and lyse tumor cells in tumor cells. At the same time, tumor cell lysis releases tumor antigen molecules and cell damage molecules recruit immune cells to reach the tumor site, reverse the immunosuppressive state of the tumor site, and produce anti-tumor effects.

Tumor cells with defective apoptosis pathways may support increased viral replication. Elevated AKT expression in tumor cells is associated with anti-apoptotic mechanisms and has been shown to facilitate the replication of some viruses. Pharmacological and genetic inhibition of PI3K (AKT upstream protein) or Akt resulted in a significant decrease in vaccinia virus production (from 80% to >/=90%) (27). (**Figure 1**).

This progress underscores the potential of oncolytic viruses as a targeted cancer therapy, leveraging specific vulnerabilities in tumor cells to enhance therapeutic efficacy.

### 2.2 Mechanism of action of oncolytic viruses

Oncolytic viruses (OVs) can mediate anti-tumor activity through several mechanisms (1): Direct Tumor Cell Killing: OVs replicate specifically within tumor cells, leading to immunogenic cell death (ICD) that directly destroys these cells. (2) Release of Tumor-Associated Molecules: The destruction of tumor cells by OVs results in the release of soluble tumor-associated antigens (TAAs), damage-associated molecular patterns (DAMPs), and pathogen-associated molecular patterns (PAMPs). These molecules can recruit and activate antigen-presenting cells (APCs), such as immature dendritic cells (DCs) and innate lymphoid cells, to the site of viral infection. Immature DCs capture TAAs and migrate to regional lymph nodes, where they initiate an adaptive T cell response against the tumor. (3) Enhanced Antigen Presentation: The virus-induced release of type I interferons and chemokines boosts the levels of antigen processing and presentation factors, including the expression of MHC class I molecules. This results in the recruitment of tumor-specific CD8+ T cells (cytotoxic T lymphocytes, CTLs) and NK cells, which recognize and kill tumor cells. (4) Systemic Anti-Tumor Responses: CTLs can also target distant tumor cells, including those at metastatic sites. Furthermore, the interferon response can increase the expression of immune checkpoint molecules on tumor cells, such as programmed cell death ligand 1 (PD-L1) and cytotoxic T lymphocyte-associated antigen 4 (CTLA-4). This upregulation of immune checkpoints can make tumors more susceptible to checkpoint blockade therapies following oncolytic virus treatment (28). (**Figure 1**).

These mechanisms highlight the multifaceted approach of oncolytic viruses in targeting and destroying tumor cells while enhancing the overall anti-tumor immune response.

### 2.3 Challenges of oncolytic virus

Despite the multiple advantages of oncolytic viruses (OVs) over other immunotherapies, traditional administration methods, primarily intratumoral injection, remain limited in effectiveness for deep-seated or metastatic tumors. Consequently, intravenous injection has emerged as a promising alternative and has yielded some positive results (29). However, preclinical studies have highlighted several challenges associated with intravenous administration, such as non-specific tissue uptake, neutralization by antibodies, and interactions with human blood cells (30, 31). Notably, there has been limited research on the role of neutrophils—the most abundant immune cells in peripheral blood—in relation to oncolytic viruses.

## 3 Neutrophils

### 3.1 Introduction to neutrophils

Neutrophils are the most abundant white blood cells in human peripheral blood, roughly 60% of peripheral blood leukocytes. Characterized by their multi-lobed nuclei, neutrophils originate from medullary precursors in the bone marrow. They undergo a series of developmental stages—from myeloblasts to promyelocytes, myelocytes, metamyelocytes, band neutrophils, and finally segmented neutrophils—before being released into the peripheral circulation (32, 33). This maturation process is regulated by various transcription factors, including PU.1 and CCAAT/enhancer-binding proteins (C/EBP). Neutrophil production is robust, with daily output reaching up to 5 × 10^10–10 × 10^10 cells, highlighting their crucial role in the innate immune system (34).

### 3.2 Neutrophils in cancer

In cancer, neutrophils have a dual role (35). They can promote tumor angiogenesis, thereby aiding tumor growth and progression. The neutrophil-to-lymphocyte ratio (NLR) serves as a marker of systemic inflammation (36, 37) and is associated with various malignancies, including metastatic gastric cancer (38), metastatic breast cancer (39), and triple-negative breast cancer (40). Clinical data also suggest that neutrophil expansion can influence immune suppression after cancer resection, facilitating immune escape and leading to poorer outcomes (41, 42). Additionally, neutrophils within tumors may undergo rapid and self-destructive cell death through NETs, with components like histones and neutrophil elastase promoting cancer cell proliferation, adhesion, migration, and metastasis (43).

However, neutrophils have phenotypic plasticity, and type I IFN polarizes tumor-associated neutrophils into anti-tumor N1 phenotypes in mice and humans (44) and TGFβ-regulated neutrophils exhibit a unique N1 profile (45).

### 3.3 Antiviral effect of neutrophils

Traditionally, neutrophils are recognized for their critical role in responding to bacterial and fungal infections as the first immune cells to arrive at sites of injury and infection. Their clearance mechanisms are well understood. While antiviral responses have traditionally been attributed to T cells and B cells, recent evidence reveals that neutrophils, as innate immune cells, also play a significant role in combating viral infections.

Neutrophils act as the first line of defense by engaging in various immune activities (46). They clear pathogens through interactions with other immune cells, direct phagocytosis (47), and the release of cytokines, chemokines, and antimicrobial components (48). Additionally, neutrophils can eliminate viruses through Toll-like receptor (TLR)-mediated formation of neutrophil extracellular traps (NETs). Electron microscopy, radioactivity, and fluorescence analyses have demonstrated that neutrophils exhibit phagocytic functions similar to macrophages, effectively engulfing viruses such as influenza virus (IVA), vesicular stomatitis virus (VSV), Ebola virus, Marburg virus, and hepatitis virus. This phagocytic activity initiates antiviral processes or activates innate immunity through pattern recognition receptors (PRRs) (49).

Neutrophils produce various antibacterial and antiviral substances, including myeloperoxidase (MPO), defensins (50–52), and antimicrobial peptides (53), which have been shown to possess both antibacterial and antiviral effects. NETs, extracellular structures composed of genomic DNA, histones, defensive proteins, and proteases (54), play a crucial role in trapping and inactivating viruses. This extracellular matrix, likened to a “mosquito net,” captures viruses, and the granular proteins within the NETs contribute to virus inactivation (55).

Moreover, neutrophils can enhance antiviral responses by interacting with other immune cells, such as natural killer (NK) cells. Evidence suggests that neutrophils can activate adaptive immunity through CD8+ T cell activation following pathogen phagocytosis (56).

We have summarized the dual role of neutrophils in tumor and its antiviral mechanism. However, the relationship between neutrophils and a special class of viruses that are used as tumor therapeutic agents is not clear.

## 4 Neutrophils in oncolytic virus therapy

Genetic engineering has enabled the transformation of viruses such as AdV, VACV, HSV, MV, VSV, RV, and NDV into oncolytic virus products, enhancing their selectivity and efficacy in targeting tumors (57–62), In 2005, H101, an adenovirus-based oncolytic agent, was approved in China for the treatment of cancer patients (63). Currently, numerous oncolytic virus products are undergoing preliminary animal studies and clinical trials in the quest for improved therapeutic outcomes.

Despite the promise of virotherapy, the therapeutic effects of oncolytic viruses are not always as effective as hoped. Oncolytic viruses are designed to recognize oncogenic signaling pathways that are highly expressed in tumor cells, replicate specifically within these cells, and induce the release of tumor antigens while overcoming immunosuppression in the tumor microenvironment. Traditional administration methods, primarily intratumoral (i.t.) injection, are limited in effectiveness for deep or metastatic tumors. Consequently, intravenous (i.v.) injection is being explored as an alternative. However, i.v. delivery must navigate barriers posed by immune cells in peripheral blood before reaching the tumor site (64).

Next, we summarized the content of common oncolytic virus species and neutrophil-related content.

### 4.1 Oncolytic vaccinia virus and neutrophils

As a double-stranded DNA oncolytic virus from the natural poxvirus family, Vaccinia Virus (VACV) offers several advantages over other oncolytic viruses (OVs). These include its specific targeting of tumor cells, lack of specific receptors, short life cycle, robust replication in hypoxic tumor microenvironments, and lack of integration with the host cell genome, making it a strong candidate for oncolytic virus vectors (65). Understanding the interaction between VACV and the immune system, particularly neutrophils, is crucial for the development of clinically effective VACV-based OVs. Evidence suggests that both wild-type and recombinant VACV strains can induce significant neutrophil infiltration and migration. For example, recombinant VACV expressing human interleukin-1 beta (HIL-1beta) was tested in a mouse model with subcutaneously established pancreatic tumors. Intravenous injection of this recombinant VACV led to notable tumor size reduction and a significant presence of neutrophils at the tumor site, accompanied by tumor cell necrosis (16). Similarly, in a study involving recombinant VACV expressing interleukin-2 (IL-2), direct intratumoral injection resulted in neutrophil aggregation and tumor necrosis in some patients with malignant mesothelioma (17).

Modified Vaccinia Virus Ankara (MVA) has also been shown to induce leukocyte migration, especially of neutrophils. This migration is mediated by the production of chemokine receptors such as CCR1 and CXCR2 in mouse pulmonary fibroblasts and bone marrow-derived macrophages following MVA infection, independent of Toll-like receptor 2 (TLR2) signaling. These chemokines facilitate neutrophil infiltration and inflammation, enhancing the adaptive immune response induced by MVA (66). Complement component C5 (67) further contribute to neutrophil aggregation and migration. However, the precise role of neutrophils in these processes, including their ability to engulf and neutralize viruses, remains unclear.

*In vitro* studies using VACV labeled with ^14C and ^3H demonstrated that neutrophils can phagocytose VACV, with the virus being detected in intracellular lysosomes. This process is serum-dependent, and VACV load decreases over time due to neutrophil activity (68). In murine models, both wild-type and recombinant VACV expressing tumor necrosis factor showed that while NK cells and cytotoxic T lymphocytes (CTLs) did not significantly alter VACV levels, a dramatic and transient increase in neutrophils was observed, which limited VACV replication (69). Our research group has also found that inhibiting neutrophil function can enhance the anti-tumor efficacy of oncolytic VACV (70).

Despite their role in controlling viral replication through phagocytosis, VACV has evolved mechanisms to evade the immune response. VACV Complement Control Proteins (VCPs) bind to complement components C3 and C4, and to heparin and heparan sulfate proteoglycans on the cell surface. This interaction reduces neutrophil infiltration and decreases the effectiveness of human neutrophils and NK cells, aiding VACV in escaping immune destruction (71, 72).

Additionally, neutrophils can function as antigen-presenting cells, bridging innate and adaptive immunity. After intradermal injection of MVA, neutrophils can transport the virus from the dermis to the bone marrow, aiding in the activation of CD8+ T cells (73). Moreover, a recombinant VACV strain expressing the HIV-1 C antigen, but lacking specific viral genes (A52R, K7R, and B15R), has been shown to affect the NF-kB signaling pathway in mice. This alteration enhances the antigen- presenting capabilities of neutrophils (74, 75).

Understanding these interactions between VACV, neutrophils, and the broader immune system is essential for optimizing the therapeutic potential of oncolytic viruses.

### 4.2 Vesicular stomatitis virus and neutrophils

Unlike the traditional idea that OVs targets tumor cell replication and leads to tumor cell lysis, vesicular stomatitis virus (VSV) injection reduces blood flow inside the tumor by inducing apoptosis of tumor cells, but viral replication is limited. The results of tumor transcription spectroscopy showed that viral infection caused the increase of neutrophil chemokine 1(C-X-C ligand 1, CXCL1) and chemokine 5(C-X-C ligand 1, CXCL5), and induced neutrophil infiltration into infected tumor tissues. Injection of VSV after neutrophils are pre-deleted with RB6-8C5 antibodies increases the replication and spread of VSV in tumor tissue, but also eliminates apoptosis in tumor cells that are not infected with VSV. These results suggest that excess neutrophils inhibit OVs replication and transmission, but targeted recruitment of neutrophils to infected tumor sites can enhance the killing of malignant tumor cells (76).

There is also evidence that intravenous injection of VSV can not only infect tumor cells, but also directly infect and destroy the vascular system of tumors. Three-dimensional reconstruction shows that VSV-infected tumors lack blood flow in tissues compared with uninfected tumor tissues. These results demonstrate that VSV replicates in the tumor neovascularization system and spreads within the tumor mass, triggering an inflammatory response and forming thrombus, a process that forms dependent on the presence of neutrophils. After deletion of neutrophils with anti-GR-1 monoclonal antibodies, infected tumors showed significantly reduced fibrin deposition and reduced thrombosis, demonstrating that neutrophils are necessary to induce tumor perfusion loss during VSV infection of tumor tissue (77).

At the same time, there has been evidence that bone marrow, blood, lung and spleen were collected by intravenous injection of VSV with 1×10^9 PFU for 3h and 24h, and acute changes of neutrophils during infection were analyzed by flow cytometry. VSV infection resulted in rapid migration of neutrophils from bone marrow to lung accumulation. The accumulation of immature neutrophil antigen presenting potential in the spleen is also increased. In addition, infection with VSV labeled with green fluorescent protein (GFP) revealed the potential of neutrophils to acquire the protein encoded by the virus transgene. After incubating spleen cell populations with αCD3 and αCD28 *in vitro*, a significant proportion of neutrophils became GFP positive. This suggests that neutrophils are able to take up VSV or VSV is able to infect neutrophils after VSV infection (78).

These findings offer new insights into the role of neutrophils in the antitumor activity associated with vesicular stomatitis virus (VSV). Neutrophils recruited by VSV enhance cytotoxicity against tumor cells. However, an excess of neutrophils may inhibit both the replication and dissemination of VSV. These results indicate that the involvement of neutrophils should be carefully considered in all aspects when utilizing VSV for future therapeutic applications.

### 4.3 Adenovirus and neutrophils

Adenovirus (Adv) is a non-enveloped, double-stranded DNA virus from the adenovirus family. It is used as a vector for vaccines against viruses like Ebola (79) and SIV (which causes AIDS in apes). Preclinical trials of the Adv-based SIV vaccine have demonstrated that it can induce a strong neutrophil response and activate neutrophils, which exhibit both phenotypic and functional changes. This activation leads to B cell activation and antibody production, primarily influenced by post-infection neutrophils, and is independent of Interleukin-10 (IL-10). Thus, neutrophils contribute to both innate and adaptive immunity in Adv vector vaccine infections (80).

Despite the potential of oncolytic Adv immunotherapy, there is a lack of specific biomarkers for its effectiveness. Elevated levels of Interleukin-8 (IL-8) in many cancers have been associated with poor outcomes in oncolytic Adv therapy. This suggests that IL-8 may influence the efficacy of oncolytic Adv therapy, IL-8 blockade together with adenovirus can influence TIL proliferation and activation when co-cultured with TANs isolated from ovarian tumors (81).

In clinical data from 290 patients treated with oncolytic Adv between 2007 and 2012, the use of Adv modified with granulocyte-macrophage colony-stimulating factor (GM-CSF) improved patient prognosis (hazard ratio (HR) 0.378, p < 0.001). Patients with a lower neutrophil-to-lymphocyte ratio before treatment had longer overall survival (p < 0.001). These findings provide insights into optimizing oncolytic Adv therapy and patient selection (14).

In addition to direct interactions between neutrophils and oncolytic Adv, neutrophil-related proteins and peptides have also been implicated. Human Neutrophil Peptides (HNPs), such as HNP-1, HNP-3, and HNP-4, have been shown to play a protective role in respiratory diseases caused by Adv. ELISA results indicated increased levels of these peptides and an associated rise in neutrophil count, suggesting an anti-Adv immune effect (82). This results in increased production of pro-inflammatory cytokines, such as tumor necrosis factor (TNF) and Human Macrophage Inflammatory Protein 2 (MIP-2), enhancing the anti-tumor effects of recombinant oncolytic Adv (83).

Due to the limited literature, we have summarized some aspects of the relationship between neutrophils and oncolytic adenovirus in this content. While we did not delve into the direct interaction between neutrophils as immune cells and oncolytic adenoviruses, our summary provides valuable insights that may guide future clinical use of Adv in tumor therapy.

### 4.4 Herpes simplex virus and neutrophils

Herpes Simplex Virus (HSV) is a double-stranded DNA virus with several strains, such as HSV-1 and HSV-2, that exhibit oncolytic properties. Among these, HSV-1 has been the most commonly modified for oncolytic therapy. For example, Talimogene laherparepvec (T-Vec), an HSV-1 derivative, is used for treating melanoma (84). In studies involving Vaccinia Virus (VACV), neutrophils have been shown to phagocytose viruses, and similar evidence has been observed for HSV-1. Puncture biopsies and electron microscopy have demonstrated that HSV-2 virions and viral capsids can be found in neutrophils from genital infections, confirming that neutrophils phagocytose HSV-2 and play a role in limiting its replication and clearance (85, 86).

In studies of delayed hypersensitivity (DTH) after HSV-1 infection in BALB/c mice, neutrophils were among the first immune cells to arrive at the infection site (87). Their presence significantly inhibited HSV-1 replication, indicating that neutrophils are crucial in the DTH response. They are recruited to the site by human macrophage inflammatory protein-1 alpha (MIP-1α) and activated by interleukin-1α, which helps inhibit viral replication (88).

*In vitro* studies with neutrophils from neonates and adults co-cultured with HSV-infected Vero or CEM tumor cells showed that neutrophils significantly reduced HSV’s ability to form plaques (89).

In studies involving HSV-2 carrier oncolytic viruses, such as the FusOn-H2 strain with a deleted N-terminal region of the ICP10 gene, neutrophils were found to lyse tumor cells effectively. FusOn-H2 exhibited oncolytic effects in 80% of tumor cell lines *in vitro*, and the remaining 20% resistant lines were also susceptible *in vivo*. After injecting FusOn-H2 into mouse tumors and analyzing neutrophils from the treated tissues, it was found that neutrophils in virus-infected tumors had a higher ability to lyse tumor cells compared to those in untreated tumors. These neutrophils also showed increased cell migration. This evidence underscores the potential of neutrophils to enhance the anti-tumor effects of the HSV-2 carrier oncolytic virus FusOn-H2 (90).

These findings highlight that neutrophils interact with both HSV-1 and HSV-2, contributing to antiviral immunity. However, it is important to note that HSV-1 rapidly absorbs into the skin after infecting the epidermis of mice. Treatment with anti-LY6G-specific monoclonal antibodies induces systemic neutropenia but does not affect virus replication or damage development. Instead, Gr-1(+) cells seem to limit viral replication (91). Interestingly, enzyme-linked immunosorbent assay (ELISA) revealed that HSV-1 enhances the expression of the cell death receptor Fas and its ligand FasL on neonatal neutrophils, inducing apoptosis. However, this effect was less pronounced in adult neutrophils (92).

### 4.5 Measles virus and neutrophils

Measles virus (MV) is a single-stranded, negative-sense RNA virus with an envelope, belonging to the Paramyxoviridae family. MV targets tumor cells through various receptors, including lymphocyte activation molecule 150 (CD150), lymphocyte activation molecule 46 (CD46), and Nectin cell adhesion molecule 4 (NECTIN-4). Tumor cells often express higher levels of CD46 compared to healthy cells, which enhances MV’s specificity for tumors. However, the widespread use of MV vaccines presents a challenge for MV-based oncolytic therapies, as the immune system can clear the virus quickly after injection (25).

Research has shown that both wild-type MV (WT-MV) and tumor-lytic vaccine strains such as MV-Vac can infect and replicate within neutrophils, resulting in increased survival of these cells post-infection. MV-Vac, in particular, activates neutrophils more effectively than WT-MV by inducing new RNA and protein synthesis. This activation stimulates the secretion of anti-tumor cytokines such as IL-8, MCP-1, and IFN-alpha, and triggers the release of TRAIL (TNF-related apoptosis-inducing ligand), enhancing the anti-tumor effect. Although neutrophils are not the sole factor influencing viral replication, they play a critical role in the anti-tumor efficacy of oncolytic MV (93). Additionally, in a mouse model with congenital immune deficiencies, subcutaneous inoculation of tumor cells followed by MV-Vac therapy demonstrated that neutrophils are crucial for tumor regression.

In studies involving two different B-cell malignancy models, a recombinant oncolytic MV expressing human granulocyte colony-stimulating factor (HG-CSF) was evaluated for its effects on the MV oncolytic response. While simultaneous treatment with MV-hG-CSF was observed, neutropenia reduced the oncolytic effect of MV-hG-CSF in one model, specifically the Nalm-6 human acute B-lymphocytic leukemia cell line (15).

Another study involved the use of recombinant MV expressing mouse granulocyte-macrophage colony-stimulating factor (GM-CSF) in a human lymphoid tumor model using immunodeficient mice. The study compared the effects of parental MV, ultraviolet-irradiated MV, and MV-GM-CSF. Results indicated that intratumoral injection of MV could reduce or eliminate tumor progression, with MV-GM-CSF further enhancing the oncolytic effect. This enhancement was attributed to neutrophil infiltration and the absence of NK cells and macrophages in the tumor. The strong neutrophil response was closely linked to tumor regression (94). Further research showed that recombinant MV can stimulate a potent neutrophil-mediated anti-tumor response, which is enhanced by cytokines to boost the anti-tumor activity of neutrophils (95).

These findings suggest that the role of neutrophils may vary across different models during MV-based oncolytic therapy. Besides neutrophils, Helicobacter pylori neutrophil-activating protein (NAP) also plays a significant role in treating metastatic breast cancer using MV as a vector. Recombinant MV strains, such as MV-lambda-NAP and MV-s-NAP, which secrete NAP, have been shown to improve the median survival rate of metastatic breast cancer patients. This improvement is associated with increased levels of Th1-type cytokines, which further enhance the anti-tumor effects of oncolytic MV (96, 97).

## 5 Conclusions and perspectives

Neutrophils have traditionally been recognized for their role in combating bacterial and fungal infections through various mechanisms. However, emerging evidence highlights their integral role in antiviral immune responses, especially as the first immune cells to arrive at the site of infection following viral exposure. Oncolytic virus (OV) therapy, a promising approach in cancer treatment, presents a unique challenge in understanding neutrophils’ dual roles in antiviral and anti-tumor responses.

**Figure 2:**
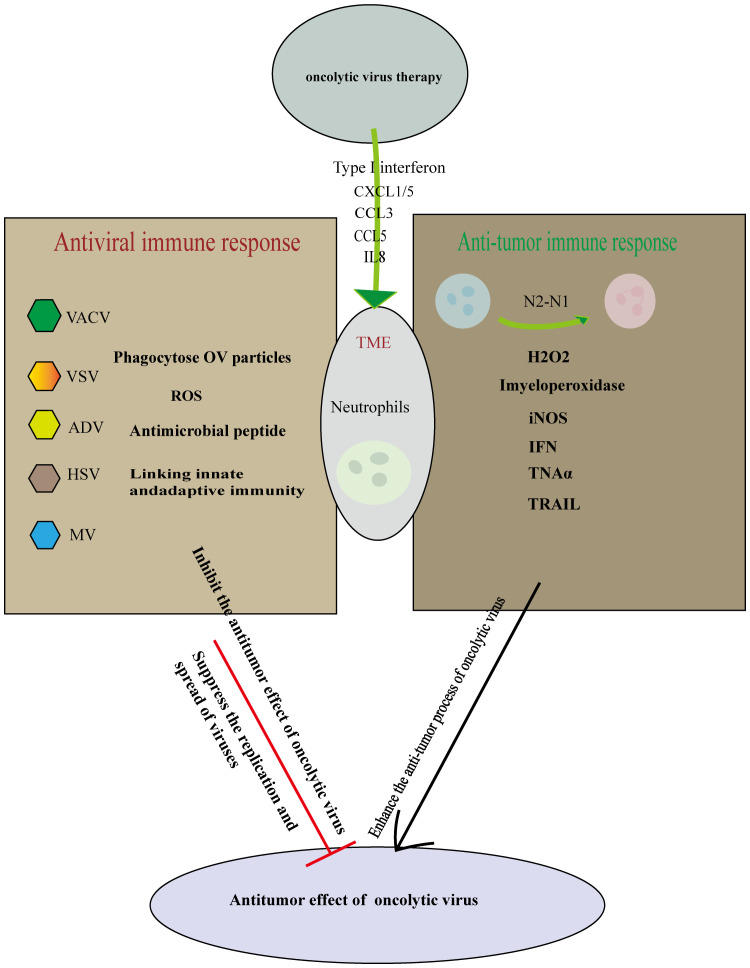
Cutting both ways: Neutrophils in oncolytic virus immunotherapy. Oncolytic virus replicates and lyses tumor cells specifically, causing immune death of tumor cells and releasing cell damage factors and tumor antigen molecules. On the one hand, the oncolytic virus infection induces tumor-associated neutrophils anti-tumor phenotype differentiation from N2-N1, Initiate anti-tumor action and mediate tumor cell killing. On the other hand neutrophils can use multiple mechanisms to perform antiviral effects. Therefore, the role of neutrophils should be considered in many aspects in the treatment of oncolytic virus, so that the synergistic anti-tumor effect of immune cells and oncolytic virus is the strongest.

During OV therapy, neutrophils exhibit seemingly contradictory behaviors. While they can inhibit OV replication and engage in antiviral activities by recruiting cytokines and other immune factors, they also play a role in modulating the tumor microenvironment. The immunosuppressive nature of the tumor microenvironment can potentially be alleviated by neutrophil activation, thereby enhancing the anti-tumor effects of OVs (98) (**Figure 2**).

These complex interactions underscore the need for further research to reconcile these contradictory roles. Future studies should focus on finding a balance between inhibiting neutrophil activity to increase OV replication in tumor cells and subsequently activating neutrophils to counteract the immunosuppressive tumor microenvironment. Achieving this balance could optimize the anti-tumor efficacy of OVs in clinical settings.
